# Supplementary material for: PPAR-α Insufficiency Enhances Doxorubicin-Induced Nephropathy in PPAR-α Knockout Mice and a Murine Podocyte Cell Line
Source: Cells. 2024 Aug 28;13(17):1446. doi: 10.3390/cells13171446 (PMC11394432; doi:10.3390/cells13171446)
Supplement: Supplementary file 1 [file cells-13-01446-s001.zip › cells-3160343-supplementary.pdf]

Supplementary Figures S1-S10

PPAR-α Insufficiency Enhances Doxorubicin-induced Nephropathy in PPAR-α Knockout Mice and a Murine Podocyte Cell Line

Kohei Matsuta[1], Kazuko Kamiyama[1], Toru Imamoto[1], Izumi Takeda[1], Shinya Masunaga[1], Mamiko Kobayashi[2], Naoki Takahashi[2], Kenji Kasuno[2], Masanori Hara[3], Masayuki Iwano[2], Tadashi Toyama[1,2], Hideki Kimura[1,2]

[1]Department of Clinical Laboratory, University of Fukui Hospital, Fukui, Japan

[2]Division of Nephrology, Department of General Medicine, School of Medicine, Faculty of Medical Sciences, University of Fukui, Fukui, Japan

[3] Iwamuro Health Promotion Center, Niigata, Japan

Supplementary Figure S1

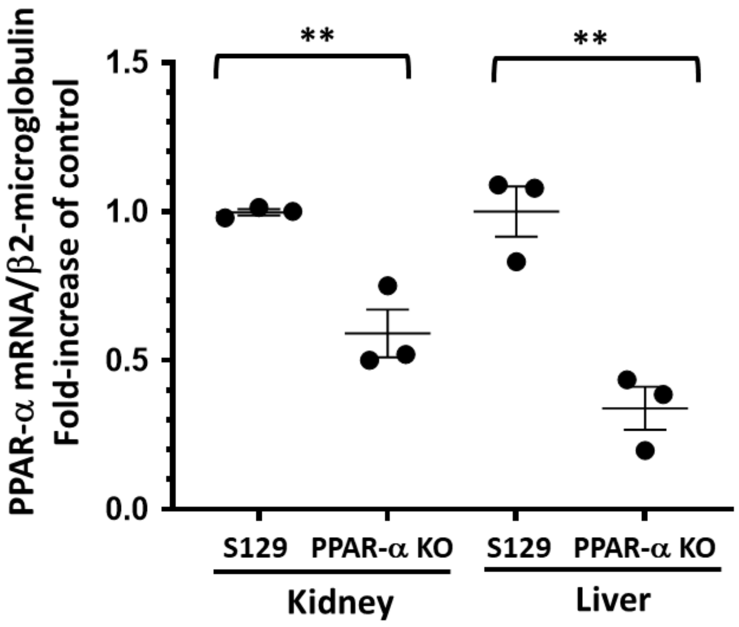

Supplementary Figure S1. Expression of PPAR-α mRNA in the kidneys and liver of Wild-type S129 (WT) and PPAR-α knockout S129 (PAKO) mice during feeding.

The mRNA levels of PPAR-α and β2-microglobulin of the kidneys and liver of WT and PAKO mice during feeding were determined by real-time RT-PCR assay and normalized to those of β2-microglobulin. The average levels of PPAR-α mRNA of each of kidneys and liver in the WT mice were set to 1.0, respectively. Individual data are expressed as dot plots with mean ± SEM of a representative experiment (n= 5). **, P<0.01, different between the indicated groups according to unpaired Student's t-test.

Supplementary Figure S2

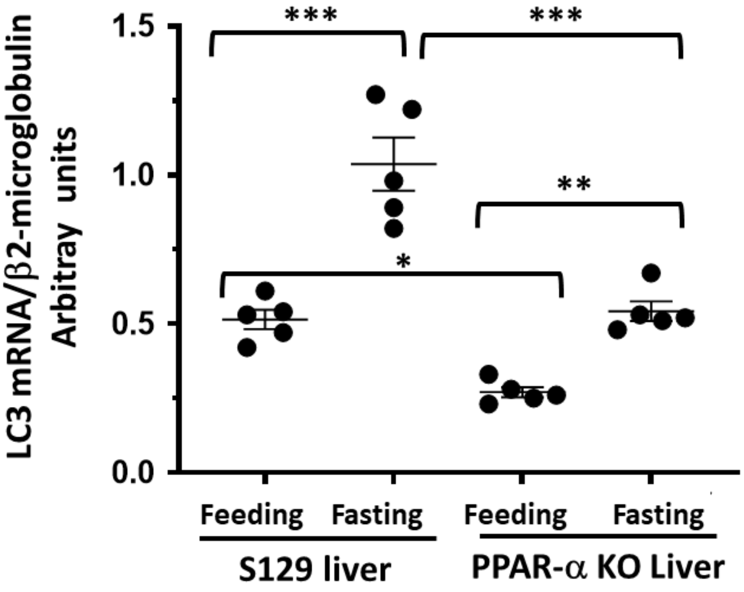

Supplementary Figure S2. Expression of LC3 mRNA in the liver of Wild-type S129 (WT) and PPAR-α-knockout S129 (PAKO) mice during feeding and fasting.

The mRNA levels of LC3 and β2-microglobulin in the liver of fed WT and PAKO mice and 48-hour fasted WT and PAKO were measured by using real-time RT-PCR assay and normalized to that of β2-microglobulin mRNA. The mRNA levels of each sample were calculated as arbitrary units. Individual data are expressed as dot plots with mean ± SEM of a representative experiment (n= 5). *, $P<0.05$, **, $P<0.01$, and ***, $P<0.001$, different between the indicated groups according to one-way ANOVA with Tukey's multiple comparisons test.

Supplementary Figure S3

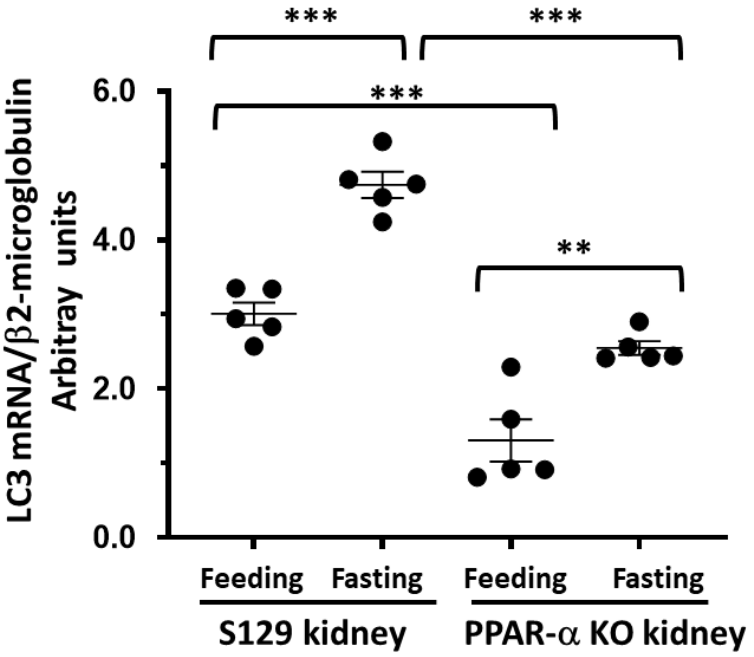

Supplementary Figure S3. Expression of LC3 mRNA in the kidneys of Wild-type S129 (WT) and PPAR-α-knockout S129 (PAKO) mice during feeding and fasting.

The mRNA levels of LC3 and β2-microglobulin in kidneys of the fed WT and PAKO mice and 48-hour fasted WT and PAKO were measured using real-time RT-PCR assay and normalized to those of β2-microglobulin. The mRNA levels of each sample were calculated as arbitrary units. Individual data are expressed as dot plots with mean ± SEM of a representative experiment (n= 5). **, P<0.01, and ***, P<0.001, different between the indicated groups according to one-way ANOVA with Tukey’s multiple comparisons test.

Supplementary Figure S4

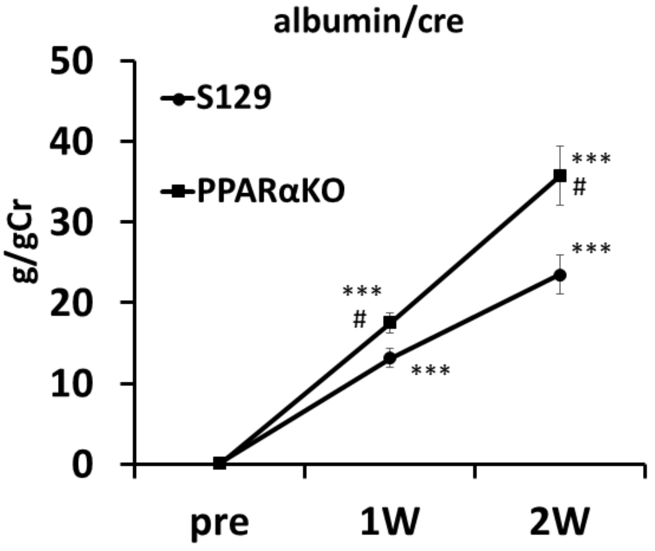

Supplementary Figure S4. Urinary levels of albumin at weeks 1 and 2 of Doxorubicin **(**DOX)-treated Wild-type S129 (WT) and PPAR-α-knockout S129 (PAKO) mice.

Urinary levels of albumin (μg/mL) and creatinine (mg/dL) at day 0 and 1- and 2-weeks post-treatment from DOX-treated WT and PAKO mice were determined by standard immunological and enzymatic assays, respectively, and normalized to creatinine levels. The results were calculated as a value of g/gCr. Individual data are expressed as dot plots with the mean ± SEM of a representative experiment (n = 6). *, $P<0.05$ and **, $P<0.01$, different between the indicated groups according to unpaired Student's t-test. #, $p < 0.05$: difference between the two groups at the same time point; ***, $p < 0.001$: difference from day 0 (pre) in each mouse group, based on unpaired Student's *t*-test.

Supplementary Figure S5

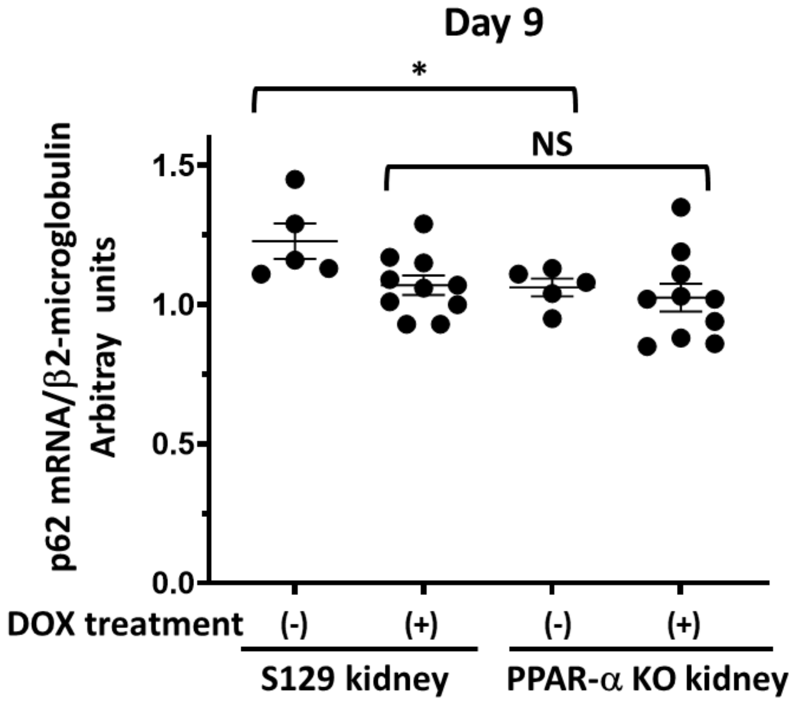

Supplementary Figure S5. Expression of p62 mRNA in the kidneys of Wild-type S129 (WT) and PPAR-α-knockout S129 (PAKO) mice with and without Doxorubicin (DOX) treatment.

The mRNA levels of p62 and β2-microglobulin in kidneys of DOX-untreated WT and PAKO mice and DOX-treated WT and PAKO mice at day 9 were measured by using real-time RT-PCR assay and normalized to those of β2-microglobulin. The mRNA levels of each sample were calculated as arbitrary units. Individual data are expressed as dot plots with mean ± SEM of a representative experiment (n = 5–10). NS is not significantly different; *, $P<0.05$, the indicated groups differ according to one-way ANOVA with Tukey's multiple comparisons test.

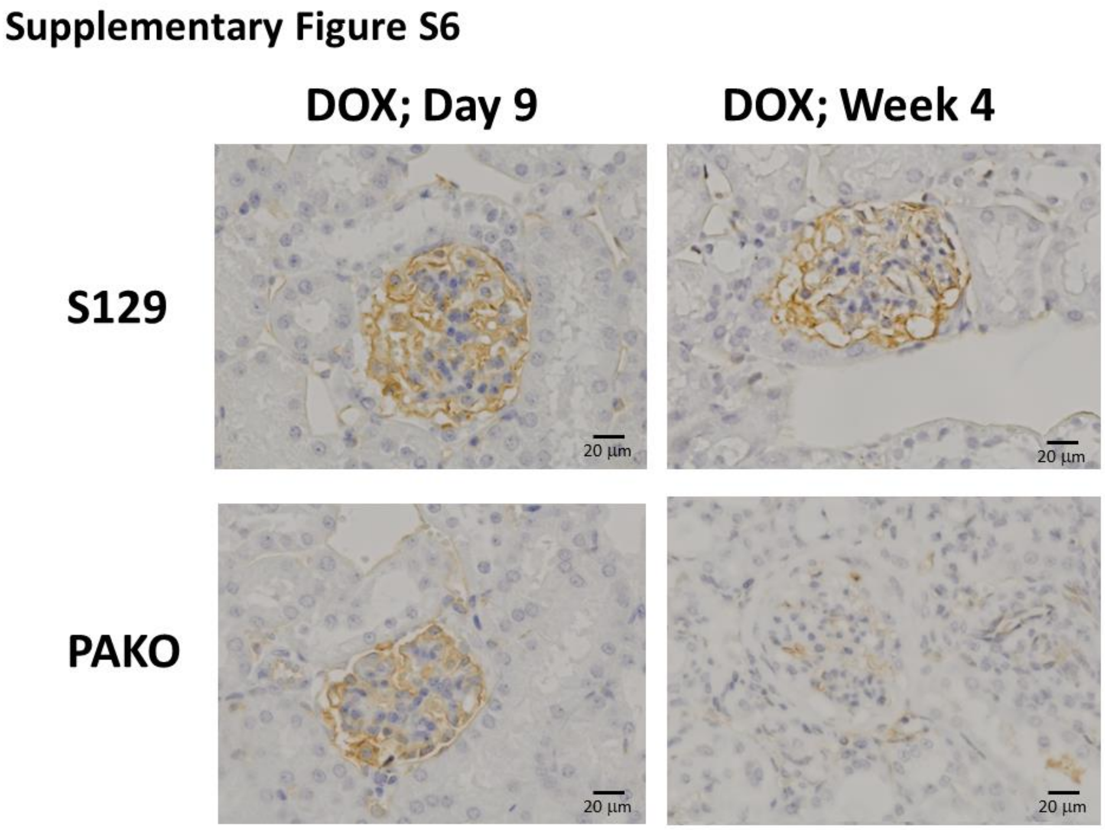

Supplementary Figure S6. Podocalyxin immunostaining of renal tissues at day 9 and week 4 from Doxorubicin (DOX)-treated Wild-type S129 (WT) and PPAR-α-knockout S129 (PAKO) mice.

Male PAKO S129 and WT S129 mice (10–12 weeks old, n = 6–8) were treated with DOX and sacrificed at day 9 and 4 weeks post-treatment to collect kidney samples. Podocalyxin immunostaining was performed in kidney samples (n=2 for each mice grop) at day 9 and 4-weeks post-treatment as described in the Methods section. Representative immunostaining of podocalyxin in the kidneys from DOX-treated WT and PAKO mice are shown. DOX-treated PAKO mice presented significantly lower intensity of podocalyxin staining than DOX-treated WT mice at 4-weeks post-treatment, while the two strains showed a similar intensity at day 9.

Supplementary Figure S7

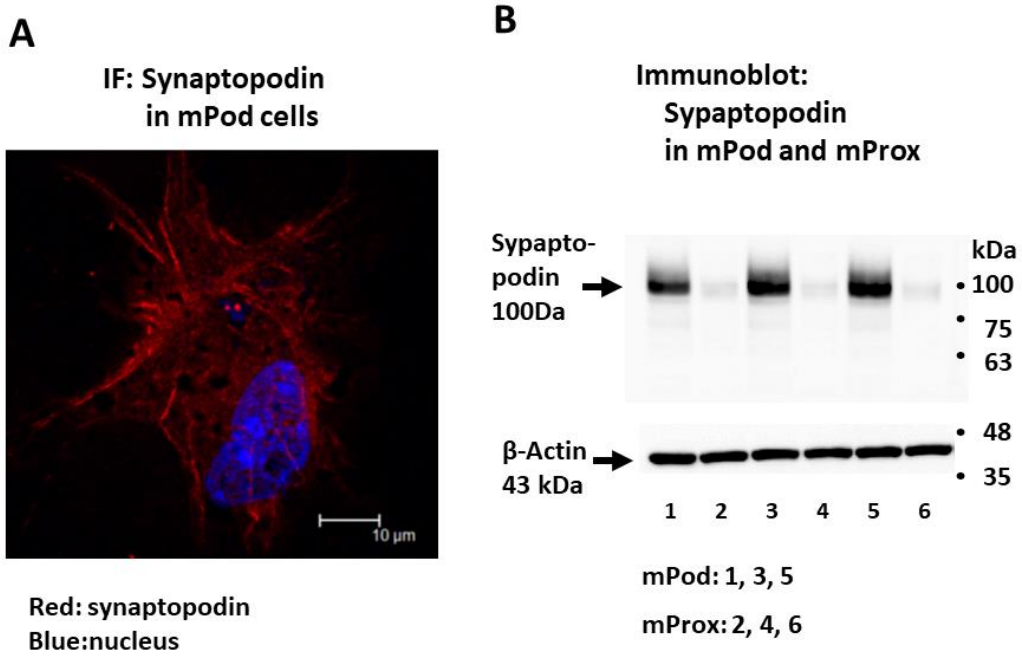

Supplementary Figure S7. mPod cells display morphological features characteristic of primary podocytes and expressed synaptopodin specific for podocytes.

(**A**) Indirect immunofluorescence was performed on mPod cells cultured on collagen I-coated chamber slides (AGC TECHNO GLASS, CO., LTD, Tokyo, Japan) fixed in 4% paraformaldehyde containing 2% sucrose. Cells thus treated were stained with mouse monoclonal anti-synaptopodin antibody (1:500 dilution; sc-515842, Santa Cruz Biotechnology Inc., Texas, USA). Alexa Fluor-647 anti-mouse monoclonal antibody (ab150115, Abcam, UK) was used as a secondary antibody. Confocal microscopy analysis was performed using a Zeiss LSM 5 Pascal Model Confocal Microscope (Carl Zeiss International, Jena, Germany). Nuclear staining was performed using Hoechst 33258.

(**B**) As described in the Methods section, mPod and mProx cells were grown semi-confluently. Amounts of synaptopodin in the cell lysates from the two different cells were determined by immunoblot analysis. mPod cells expressed considerably large amounts of synaptopodin, while mProx cells expressed only faint amounts.

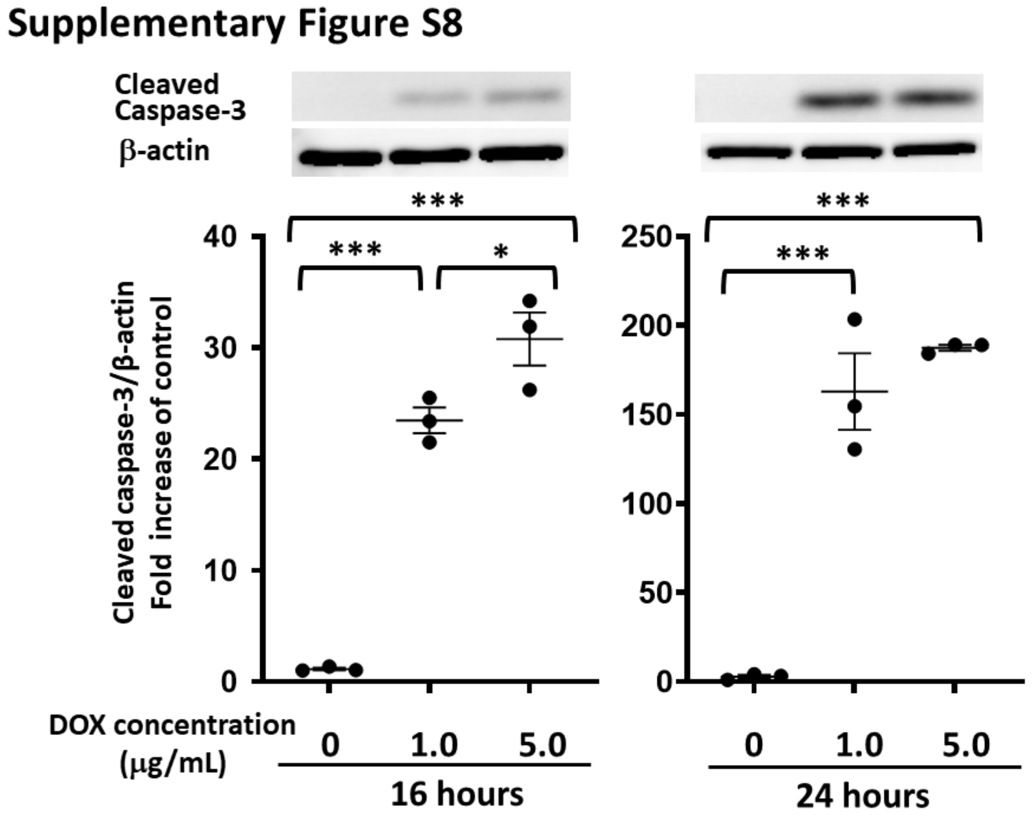

Supplementary Figure S8. Doxorubicin (DOX) increases amounts of cleaved caspase-3 time- and dose-dependently in mPod cells.

mPod cells cultured on 12-well plates coated with collagen type I were treated at 37°C for 24 h with the maintaining medium containing DOX (0, 1.0, and 5.0 μg/mL) for 16 hours or 24 hours. The amounts of phosphorylated cleaved caspase-3 and β–actin were determined by immunoblot analysis using whole cell lysates. The amounts of cleaved caspase-3 were normalized to that of β-actin. Individual data are expressed as dot plots with the mean ± SEM of a representative experiment (n = 3). *, *P*<0.05, ***, *P*<0.001, different between the indicated groups according to one-way ANOVA with Tukey's multiple comparisons test.

Supplementary Figure S9

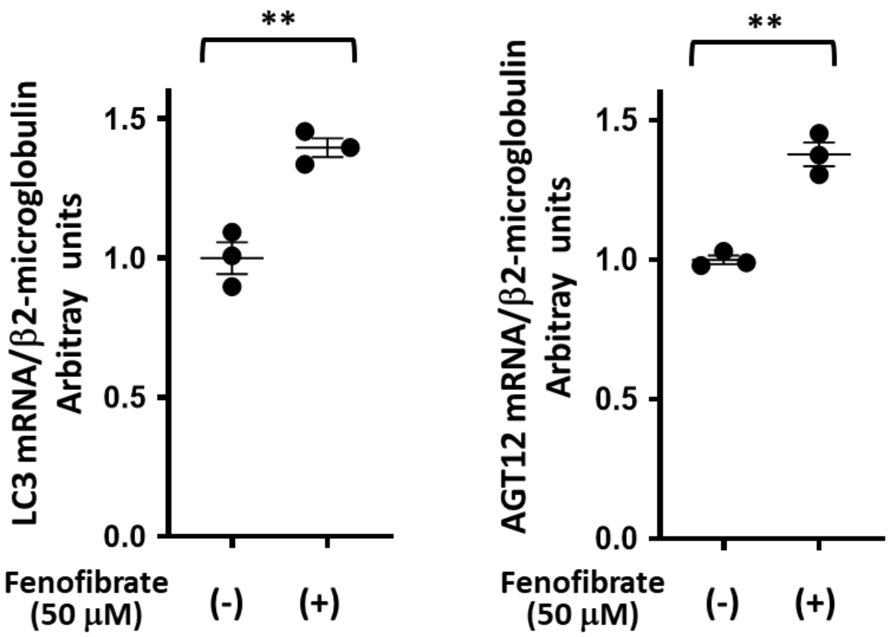

Supplementary Figure S9. Twenty-four-hour treatment with fenofibrate produces about a 1.5-fold increase in mRNA levels of LC3 and AGT in mPod cells.

mPod cells cultured on 12-well plates coated with collagen type I were treated at 37°C for 24 h with the maintenance medium (RPMI) containing fenofibrate (0 or 50 μM) for 24 hours. The mRNA levels of LC3, ATG12, and β2-microglobulin were determined by using real-time PCR assays and normalized to those of β2-microglobulin. The average mRNA levels in mPod cells untreated with fenofibrate were set to 1.0. Individual data are expressed as dot plots with the mean ± SEM of a representative experiment (n = 3). **, P<0.01, different between the indicated groups according to unpaired Student's t-test.

Supplementary Figure S10

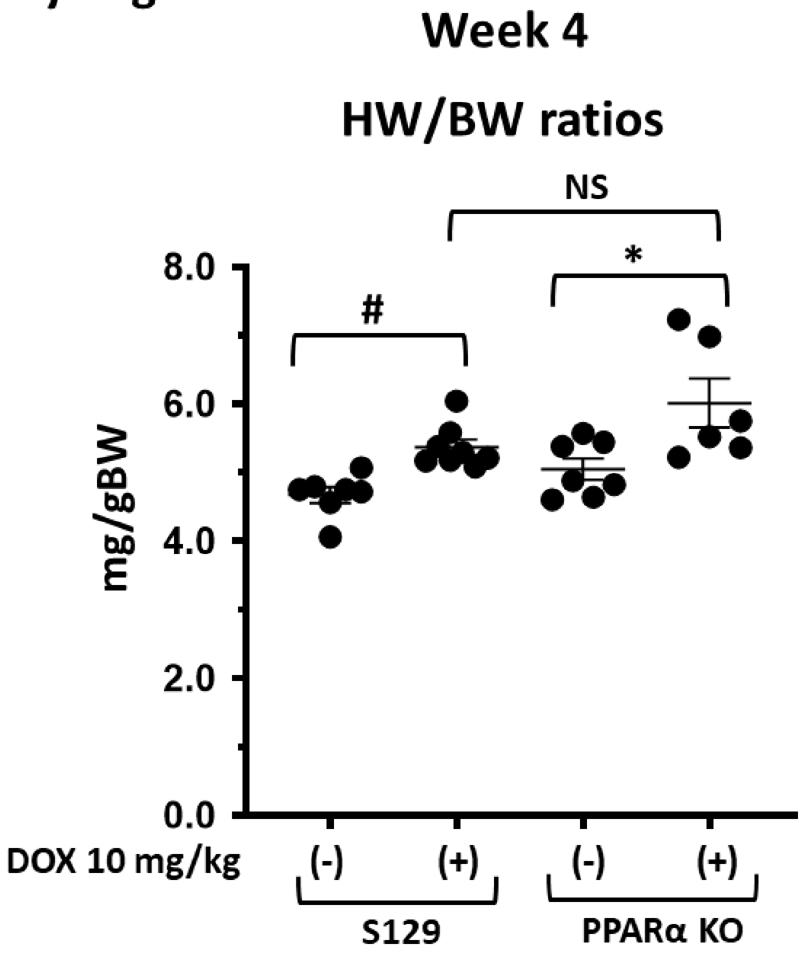

Supplementary Figure S10. Heart weight to Body weight ratios at Week 4 of Wild-type S129 (WT) and PPAR-α-knockout S129 (PAKO) mice with and without Doxorubicin (DOX) treatment.

Male PAKO S129 and WT S129 mice (10–12 weeks old, n = 6–8) were treated with DOX and sacrificed at 4 weeks post-treatment to collect heart samples. Heart weight (HW) and body weight (BW) were measured in each mouse of four mice groups. Heart weight to body weight ratios (HW/BW ratios, mg/gBW) were calculated as a heart weight marker and compared among the four mice groups. Individual data are expressed as dot plots with the mean ± SEM of a representative experiment (n = 6-8). NS, not significant; $^{\#}p < 0.06$, $^{*}p < 0.05$, different between the indicated groups according to one-way ANOVA with Tukey's multiple comparisons test.
